# Nurses’ Experience With Health Information Technology: Longitudinal Qualitative Study

**DOI:** 10.2196/medinform.8734

**Published:** 2018-06-26

**Authors:** Inga M Zadvinskis, Jessica Garvey Smith, Po-Yin Yen

**Affiliations:** ^1^ Riverside Methodist Hospital OhioHealth Columbus, OH United States; ^2^ Helene Fuld Health Trust National Institute for Evidence-Based Practice in Nursing and Healthcare College of Nursing The Ohio State University Columbus, OH United States; ^3^ Department of Biomedical Informatics College of Medicine The Ohio State University Columbus, OH United States; ^4^ Institute for Informatics Department of Medicine Washington University St Louis, MO United States; ^5^ Goldfarb School of Nursing Barnes Jewish College BJC Healthcare St. Louis, MO United States

**Keywords:** health IT, electronic health record, barcode medication administration, qualitative research, adaptation

## Abstract

**Background:**

Nurses are the largest group of health information technology (HIT) users. As such, nurses’ adaptations are critical for HIT implementation success. However, longitudinal approaches to understanding nurses’ perceptions of HIT remain underexplored. Previous studies of nurses’ perceptions demonstrate that the progress and timing for acceptance of and adaptation to HIT varies.

**Objective:**

This study aimed to explore nurses’ experience regarding implementation of HIT over time.

**Methods:**

A phenomenological approach was used for this longitudinal qualitative study to explore nurses’ perceptions of HIT implementation over time, focusing on three time points (rounds) at 3, 9, and 18 months after implementation of electronic health records and bar code medication administration. The purposive sample was comprised of clinical nurses who worked on a medical-surgical unit in an academic center.

**Results:**

Major findings were categorized into 7 main themes with 54 subthemes. Nurses reported personal-level and organizational-level factors that facilitated HIT adaptation. We also generated network graphs to illustrate the occurrence of themes. Thematic interconnectivity differed due to nurses’ concerns and satisfaction at different time points. Equipment and workflow were the most frequent themes across all three rounds. Nurses were the most dissatisfied approximately 9 months after HIT implementation. Eighteen months after HIT implementation, nurses’ perceptions appeared more balanced.

**Conclusions:**

It is recommended that organizations invest in equipment (ie, wireless barcode scanners), refine policies to reflect nursing practice, and improve systems to focus on patient safety. Future research is necessary to confirm patterns of nurses’ adaptation to HIT in other samples.

## Introduction

### Background

Health information technology (HIT or Health IT) is a broad concept that includes a variety of technologies, including computer equipment, system software, and infrastructure that records, stores, protects, and retrieves clinical, administrative, or financial information [1]. In the United States, by 2015 96% of all hospitals had adopted a certified electronic health record (EHR) [2]. An EHR is a repository of patient data that is stored and exchanged securely among multiple authorized users to support quality integrated health care [3]. While providers and patients appreciate the positive benefits of HIT through improved care [4], there are also problems with HIT [5]. HIT problems are noteworthy because they may result in delayed care, altered clinical decision-making, and modified care processes, which affect patient outcomes [5]. A few unintended adverse consequences of EHRs are incomplete information, usability issues leading to frustrating user experiences, and patient privacy breaches [6].

### Longitudinal Qualitative Studies About Clinicians’ Perceptions of HIT

A myriad of individual and organizational factors influence HIT, contributing to its complexity and multi-dimensional nature [7]. A socio-technical model offers insights for studying HIT in complex adaptive health care systems through eight dimensions, including: (1) hardware and software computing infrastructure; (2) clinical content; (3) human-computer interface; (4) people; (5) workflow and communication; (6) internal organizational policies, procedures, and culture; (7) external rules, regulations, and pressures; and (8) system measurement and monitoring [8]. Researchers have studied provider perceptions of HIT over time, describing the mixed (both positive and negative) effects of HIT. For example, a two-year prospective, longitudinal survey of attending physicians in three clinical areas experienced the change from a homegrown EHR to a vendor EHR [9]. Safety perceptions dropped during the first six months, but then began to rise [9]. Physicians reported that the EHR created additional work and their satisfaction dropped [9]. In a similarly-designed study, ophthalmologists did not report a significant change in overall job satisfaction over time (3, 7, 13, and 24 months post-EHR implementation), but they expressed concern about the EHR’s effect on interactions with patients and their ability to create quality documentation [10]. In primary care, even two years after EHR implementation, the EHR learning curve and computer knowledge remained challenging for interprofessional staff [11].

Longitudinal perceptions of EHRs have also been studied in nurses. Intensive care unit (ICU) nurses completed two cross-sectional survey questionnaires at 3 months and 12 months after EHR implementation and reported greater acceptance of the EHR at 12 months compared to 3 months [12]. In contrast, US nurses working on inpatient units within an academic medical center reported less positive attitudes toward the EHR 18 months after implementation compared to preimplementation and 6 months postimplementation [13]. For nurses, the timeline of adaptation for new HIT varies. A study conducted in Taiwan reported that nurses needed 3 months to understand the functionalities and benefits of an EHR [14]. In the United States, ICU nurses perceived the EHR as useful (through access to up-to-date information) at 3 months, but perceived that usefulness was not as relevant for acceptance at 12 months [12]. The authors explained that other EHR functionalities may have greater precedence over time to influence acceptance [12]. Positive computer attitudes are a significant predictor of fast adaptation [15]. Although studies exist regarding clinicians’ adaptation to HIT, there is insufficient qualitative evidence regarding facilitators, hindrances, and a longitudinal timeline of adaptation, especially among clinical nurses.

### Levels of Expectation Regarding HIT

In our prior study, we found that nurses’ expectations regarding HIT can be stratified from personal-level (human-computer interaction) to organizational-level (quality of care) [16]. The five levels were: (1) how easy the system is to use (ie, equipment and system), (2) nurses’ workflow and task performance, (3) collaboration within the nursing unit, (4) nurses’ communication across hospital disciplines and departments, and (5) the effects of HIT on quality of care (ie, patient safety and nurse/patient satisfaction). Each level expands from individual user concerns to the team and the broader organization, and each level increases in complexity. In early implementation, we found that HIT users may be more concerned with lower-level expectations, but the timeframe for nurses’ expectations, and thus adaptation, is unknown.

### Objective

We investigated HIT adaptation, which is, “a process of modifying existing conditions in an effort to achieve alignment” [17] involving workflow redesign, user training, and technology maintenance [18]. This was a longitudinal qualitative study that explored medical-surgical nurse perceptions of HIT implementation over time. We interviewed nurses three times after EHR and bar code medication administration (BCMA) implementation to capture evolving adaptation of their perceptions and behaviors in this specific job role. The objective of the study was to explore nurses’ experience of HIT implementation, and how they adapted their perceptions and behavior to HIT upgrades and optimization over time.

## Methods

### Overview

We conducted a phenomenological qualitative study at a large Midwestern academic medical center. The medical center implemented a customized commercialized EHR system (EPIC platform [19]) in October 2011, which included computerized provider order entry, electronic charting, and BCMA. Prior to 2011, a few nursing units (ie, critical care) used some electronic documentation. In 2011, all nursing units within the medical center began using the new EHR system. In this study, participants were from a medical-surgical unit that used paper charts prior to implementation.

### Sample

We used purposive sampling to recruit staff nurses who worked on a medical-surgical unit that used EHRs and BCMA and had a minimum of two years of working experience in the organization. We approached participants either face-to-face or via email and hosted private face-to-face interviews in a location away from the clinical area to ensure privacy and avoid disruption.

### Data Collection

We used a semi-structured interview guide with additional probes (Multimedia Appendix 1) to clarify and discover in-depth information. Field notes were taken during and after the interviews. We interviewed participants over three time points based on convenience, including Round 1 (R1) 3 months post-EHR implementation (winter 2012), Round 2 (R2) 9 months post-EHR implementation (summer 2012), and Round 3 (R3) 18 months post-EHR implementation (summer 2013). The length of each interview ranged from 20 to 60 minutes. All interviews were audio-recorded and professionally transcribed verbatim. We also collected basic demographic information, including age, gender, position, education, and years of experience.

### Data Analysis

Three researchers (IZ, JGS, and PY) read the transcripts independently and located relevant statements in the transcripts that expressed units of meaning. The researchers generated common themes by synthesizing the meaning units. Themes reflected a general description of the nurse participants’ experience with EHRs and BCMA. The structure for the first five themes was derived from previous work that delineated confirmed nurses’ expectations ranging from personal-level to organizational-level [16]. Dimensions of the socio-technical model, such as hardware, people, workflow communication, and policies, informed coding structure during qualitative analysis. After iterative discussion and refinement, a codebook was generated. We developed a total of 61 themes: 7 at the first level and 54 at the second level. We also kept a detailed record of our codes and their definitions as we updated them over time (Multimedia Appendix 2). Each quote was classified from one to four themes, as one quote could contain multiple units of meaning (themes), which affected thematic frequency; we defined this as theme cooccurrence. We used NVivo 10 [20], a qualitative research analysis tool, for data management and analysis. In addition, we used Gephi, a graph visualization and exploration software [21], to illustrate the cooccurrence relationships among themes, where nodes represented themes and lines signified the cooccurring relationships. The network graph was illustrated in a forced layout.

### Intercoder Reliability

We assessed and established our intercoder reliability using Cohen’s Kappa. Cohen’s Kappa has been commonly used to assess intercoder reliability, with recommendations greater than 0.7 for semi-structured interviews, especially with multiple complex codes [22-24]. We coded 10% of the transcripts together to establish consensus, as experts recommend [22,24]. For each independently-coded transcript, we calculated Cohen’s Kappa by averaging the Kappas of all themes through a multiple independent coding comparison process [22,24,25]. In our analysis, two researchers (IZ and JGS) independently coded randomly selected transcripts and discussed discrepancies; a third researcher (PY) mediated the discussion to confirm final coding. After iterative discussion, we reached a Cohen’s Kappa of 0.82.

## Results

### Principal Findings

Nineteen nurses participated in the study, with some nurses participating in multiple rounds. We conducted a total of 30 interviews: 9 from R1, 11 from R2, and 10 from R3. We were unable to interview all nurses across all 3 rounds because some nurses were unavailable (ie, schedule conflicts, transferred to new positions, declined participation). Among the 9 nurses that participated in R1, 7 participated in R2, and 3 participated in R3. Eleven nurses participated in only one interview, and 8 nurses participated in more than one interview. Among the 19 nurses, 17 were female. The age of the nurses ranged from 22 to 52 years old, with 3 to 25 years of working experience, and 58% (11/19) worked the day shift. Most nurses (15/19, 79%) were Bachelor of Science in Nursing-prepared and all worked as staff nurses. All nurses owned home computers and 79% (15/19) owned smartphones. Across all rounds, nurses rated themselves an average of 4 out of 5 in computer competency, with 1 meaning *not competent* and 5 meaning *very competent*. We used information saturation to determine the number of participants.

We assembled themes into a table format to review nurse perceptions over time (Multimedia Appendix 2), categorizing them into 7 main themes with 54 subthemes. Details regarding development of the first five primary themes, E1 to E5, are described elsewhere [16]. The longitudinal approach led to the discovery of two additional themes: *adaptation* and *organizational factors*. *Adaptation* explained both internal and external resources that influenced acclimatization to technology over time. *Organizational factors* discussed communication or HIT decisions made by organizational leaders that influenced nursing work. Quote examples and frequencies can be found in Multimedia Appendix 3.

### Thematic Findings

#### E1: Nurses’ Interaction with HIT

The E1 subthemes involved *system* (software) and *equipment* components. Equipment addressed workstations on wheels (WOWs), scanners, and wires (wired mouse/scanners and electrical cords). Nurses perceived equipment negatively because of noise, occasional (battery) power loss, and challenging use in semi-private patient rooms and narrow, crowded hallways. By R3, the information technology (IT) department installed well-liked wireless BCMA scanners. System functionality concerns included lengthy login, program shutdowns during medication administration, and BCMA scanning issues. Shutdowns occurred if users forgot to plug in the WOW when not in use, leading to power loss, or sometimes for unknown reasons. Nurses reported difficult system navigation in all rounds, especially during emergency documentation. In later rounds, nurses acknowledged easier system navigation because EHR flowsheets were updated with head-to-toe organization that reflected how nurses conducted physical assessments.

#### E2: Nursing Performance Regarding Task Accomplishment

Nurses viewed documentation as time-consuming and arduous, yet thorough. Nurses valued feedback on performance from visual indicators (green and red dots) for complete or incomplete documentation. In R1 and R2, nurses thought documentation was inefficient and not streamlined because it contained elements irrelevant to their population, required too much scrolling, appeared chaotic, and took longer than paper charting. However, by R3 some nurses expressed that documentation was streamlined and efficient since the IT department had updated the EHRs to be more compact. Nonetheless, documenting rare events caused nurses stress and confusion. For instance, blood administration involved scanning multiple barcodes in a strange pattern and emergency documentation had complicated screen layouts.

#### E3: Unit-Specific Teamwork

Since all nurses experienced glitches with EHRs, they relied on nursing unit collaboration for assistance. Collaboration impacted patient safety because nurses appreciated the ability to view all records when administering medicine to other nurses’ patients. Teamwork was also associated with improved nurses’ satisfaction. Due to strong teamwork, nurses relied on one another more than IT staff for resolving system concerns.

#### E4: Interdisciplinary Teamwork

E4 themes concerned communication between departments and disciplines. Interprofessional notes helped nurses understand the care plan promoting integrated and better care. Nurses reviewed patient transfer information before arrival. Unexpectedly, some departments stopped telephone handoffs, leading to potential missed information (ie, last pain medication). Nurses identified unequal standards between departments, such as not scanning all medications or omitting parts of admission documentation. Documentation standards varied and some prescribers did not enter orders immediately after patient assessment. Unequal standards existed in the context of shifting responsibilities, such as doctors asking nurses to input their orders. This factor frustrated nurses, and they proposed standardized documentation classes and policies to improve interprofessional communication.

#### E5: Quality of Care

E5 themes related to quality of care and nurse satisfaction. Although BCMA reduced some medication errors, the potential for error remained because BCMA did not verify multi-dose medication containers (like insulin) and prescribers could still input orders incorrectly. Additional potential for errors during physical assessment documentation could occur due to the repetitive nature of electronic documentation (mouse clicking), leading to distraction and loss of attention. Patient-nurse interactions were also altered because nurses had to position their backs to patients to document on WOWs. In addition, occasionally WOWs logged nurses off or malfunctioned, leading to pain medication delays that negatively impacted care. During R1 and R2, nurses were so frustrated with learning the system that they did not appreciate potential improvements in patient care quality. However, by R3, after nurses adapted to EHRs, they frequently mentioned better quality of patient care through access to patient history, notes from all disciplines, task/documentation reminders, and improved patient safety. Perceptions of care quality improved in R3 compared to R1 and R2.

When the EHR system was first implemented, some nurses felt scared or intimidated, although eventually it met expectations or appeared that it would in the future. The most common expectation was reduced documentation that would allow for more time with patients, which did not happen, leading to disappointment. Nurses’ dissatisfaction and satisfaction were mentioned with similar frequencies in R1 and R3, although in R2 nurses were more dissatisfied than satisfied. The greatest sources of nurse dissatisfaction were equipment, system functionalities, inefficient documentation, and lengthy logins. Conversely, nurses were satisfied with BCMA error reduction, workflow simplification, patient protection, better care, and documentation thoroughness and reminders. By R3, after nurses adapted, some nurses expressed that the EHR system offered more time for higher-quality patient care.

#### Adaptation

Adaptation affected nurses’ acclimatization to new technology over time. Nurses discussed self-learning through personal motivation, practice, and long-term use. Self-learning occurred through the EHR playground, where nurses could explore the EHR layout. However, training was a major concern with rushed, fast, and overwhelming classes that were provided too far in advance of implementation. Nurses commented that training classes did not reflect nurses’ workflow; they only showed system design and navigation.

#### Organizational Factors

Organizational factors, such as policies, requirements, and decisions made by leadership, were frequently mentioned with nurses’ dissatisfaction. Clinicians expected leaders to explain rationale for HIT decisions that would impact clinical practice. For example, nurses were not aware of the rationale for policies not allowing a copy/paste function, or real-time documentation which expected nurses to chart assessments immediately after care. Nurses expected hospital leadership to be more aware of bedside nursing workflow and resolve issues quickly. Leadership added requirements or responsibilities but did not retire old/unnecessary requirements, which added to nursing work and complicated workflow. Nurses expected leadership to advocate for system features to improve nursing workflow, so that they could spend more time at the bedside.

Nurses’ suggestions for improvement grew with each round and were often related to the system and equipment. Some suggestions were incorporated by R3, such as an exact time stamp and wireless scanners. Nurses were thankful that nursing management filtered information regarding important EHR updates that affected nursing work. Nurses valued leadership’s feedback on their performance regarding percent of scanned medications, and advised leadership to be patient, remain supportive, provide resources, and answer questions.

### Visualization of Theme and Subtheme Interrelationships

**Figure 1:**
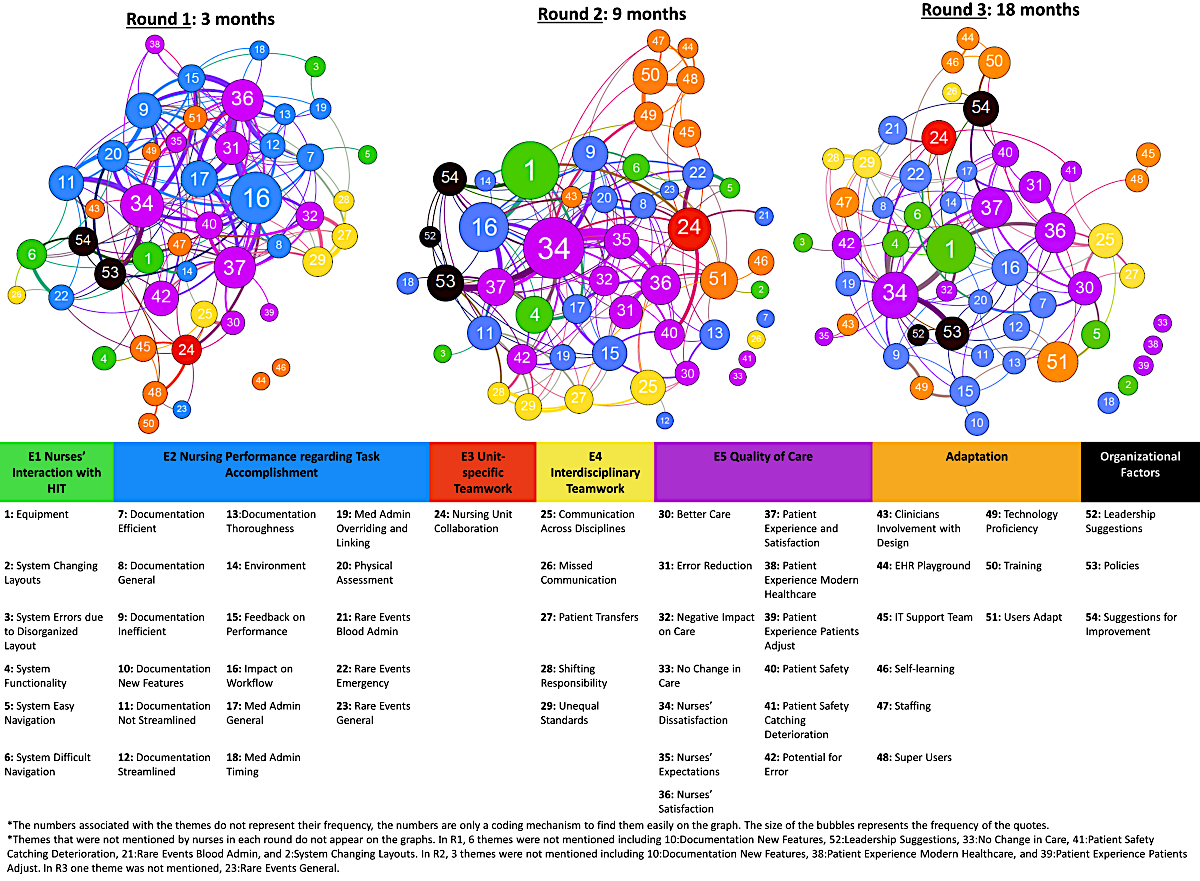
Visualization of theme and subtheme interrelationships.

We used Gephi to create a network (relationship) graph of themes for each round. Each quote contained one to four themes, while the edges/lines revealed theme cooccurrence within the same quote (Figure 1). Larger size nodes (bubbles) illustrate more frequent themes; thicker lines illustrate stronger relationships/cooccurrence. The numbers within nodes are a labeling mechanism, and do not represent the frequency of the quotes. All graphs used the same scale to facilitate thematic comparisons between rounds. Themes are organized by color, with E1 as green, E2 as blue, E3 as red, E4 as yellow, E5 as purple, adaptation as orange, and organizational factors as black.

Nurses’ dissatisfaction was related to equipment and documentation. *(#1): Equipment* was a great concern in R2 and R3 and was strongly associated with *(#34): Nurses’ dissatisfaction* in R3, as evidenced by the thick interrelationship line. *(#9): Documentation inefficient* and *(#11): Documentation not streamlined* decreased with each round, especially in R3, and they were associated with *(#34): Nurses’ dissatisfaction*. *(#35): Nurses’ expectations* of EHR strongly reflected their *(#34): Dissatisfaction* in R2.

There were trends in collaboration, communication, quality of care, and workflow. *(#24): Nursing collaboration* was greatest in R2. *(#25): Communication across disciplines* was increasingly connected to *(#30): Better care* and *(#36): Nurses’ satisfaction*. *(#36): Nurses’ satisfaction* was consistently concurrent with *(#31): Error reduction*. As nurses began to adapt to HIT, the *(#16): Impact on workflow* decreased by R3.

The longitudinal approach led to discovery of two additional themes: *adaptation* and *organizational factors*. *(#51): Users adapt* became more prominent in later rounds compared to earlier rounds. *(#53): Policies* were strongly connected to *(#34): Nurses’ dissatisfaction* in all rounds.

Due to the research approach, nearly all quotes could be related to *(#34): Nurses’ dissatisfaction* or, conversely, *(#36): Nurses’ satisfaction*. Aside from these themes, *(#1): Equipment* and *(#16): Impact on workflow* were most frequent across all three rounds. In R1, the most common themes were *(#16): Impact on workflow* and *(#37): Patient experience/satisfaction*. In R2, the most common themes were *(#1): Equipment*, *(#16): Impact on workflow*, and *#(24): Nursing unit collaboration*. In R3, *(#1): Equipment* remained as the top theme, followed by *(#37): Patient experience/satisfaction*. Themes grew and changed at each time point and were interconnected differently based on the nurses’ main concerns and satisfactions at different time points. ­­Visually, the R3 graph was the most balanced as nurses’ perceptions seemed to be equally scattered across all themes compared to R1 and R2.

## Discussion

### Overview

This study explored the trajectory of change through a qualitative analysis of nurses’ experiences after EHR and BCMA implementation at three time points: 3, 9, and 18 months. We used the socio-technical model [8] to guide our discussion. Two dimensions of the socio-technical model are not addressed (“external rules, regulations, and pressures” and “system measurement and monitoring”) because nurses did not express opinions related to these dimensions.

### Hardware and Software Computing Infrastructure

Nurses shared opinions regarding HIT equipment. Workstation preferences varied: some nurses liked working with portable workstations so they could store medications in locked drawers, while other nurses desired fixed workstations in patient rooms or portable tablets. Mobile devices may promote nurses’ ability to document at the bedside and point-of-care [26]. Nurses appreciated equipment that improved functionality, such as fingerprint login scanners and wireless input devices (scanners).

### Clinical Content

Nurses offered multiple system suggestions: they wanted illuminated new and abnormal laboratory results, parameters for holding medication (eg, blood pressure), and unacknowledged orders to be highlighted in red. Nurses suggested system recognition and display of insidious abnormal trends, such as increasing white blood cell counts or decreasing hemoglobin. Physical assessment customization, such as removal of irrelevant fields or adding fields/drop-down boxes, would improve nurses’ documentation. EHR navigation could be improved with a help sheet or search tab with a glossary or key words. A patient calendar to track scheduled tests would help prepare patients and improve workflow, communication, and possibly patient satisfaction.

### Human-Computer Interface

Clinician involvement in HIT design is a potential strategy for successful adaptation [27]. Nurses expressed frustration that clinician input was not adequately integrated into the system. Communication regarding end-user requests facilitates success [28]. Previous research indicates that when nurses were involved in refining the usability of an existing EHR, end-user satisfaction increased and nurse-sensitive quality indicators improved, including fewer catheter-associated urinary tract infections, improved documentation of the presence of pressure ulcers, and fewer restraints [29]. Nurses offered suggestions for workflow redesign and EHR modifications via email request, discussion with the Nurse Manager, and during IT meetings.

### People (Training and Peer Support)

Training and competency are sociotechnical factors that affect HIT adaptation [30]. Nurses may improve their competency and technology proficiency through practice. In this study, EHR training began months prior to implementation with nurses attending four one-hour educational training sessions. During training and early implementation, nurses were encouraged to practice documentation in the EHR playground. Although this sample of nurses rated their proficiency with technology as competent (4 out of 5), nurses working in other settings may feel less proficient or less IT-literate. Low IT literacy is a known barrier to EHR implementation [31,32], and it may be helpful to improve clinicians’ computer literacy prior to implementation. Training programs may be customized to different nurses’ learning needs to promote adaptation. Nurses viewed super users as instrumental for adapting to HIT. This finding is similar to another study in which clinicians viewed super users as supportive, familiar, and knowledgeable regarding day-to-day work [33], but different from another study that showed having super users did not contribute to meaningful HIT use [34]. Effective super users may be characterized by being proactive, providing comprehensive explanations, using positive framing, and freely sharing information [33].

### Workflow and Communication

In the socio-technical model for studying HIT, the clinical workflow involved with operating HIT systems must be consistent with internal policies and procedures [8]. Lack of congruency between policy and practice was a source of nursing frustration in this study. For example, prior to EHR implementation, nurses working 12 hours would reassess patients and write, “unchanged from previous assessment” in the paper chart, which was supported by policy. After EHR implementation, nurses had to redocument a complete physical assessment every 8 hours, because IT developers set the system for eight-hour tours of duty for nurses, which was inconsistent with practice. Therefore, numerous nurses expressed the desire for a copy and paste function, but this can inadvertently lead to clinical harm [35]. Organizations interested in using a copy and paste function may benefit from adopting best practices from the Partnership for Health IT Patient Safety [35]. Eventually, the physical documentation assessment policy in the EHR was modified to reflect nursing workflow.

### Internal Organizational Policies, Procedures, and Culture

Nurses voiced that a reduced patient load (better nurse staffing) was very helpful for adapting to the new system. During the first day of EHR implementation, medical-surgical nurses cared for only one or two patients. By R3, nurses returned to caring for four to five patients, but some nurses continued to struggle with completing documentation requirements. In the future, augmented clinical HIT (where nurse staffing decisions are based on patient volume, acuity levels, etc [36]) may become more commonplace.

A 2013 integrative review found that strong leadership ensures that the team works toward successful HIT implementation [37]. Modification of hospital policies and environment may be necessary, especially when HIT changes are directed by a vendor [38]. Leaders are expected to communicate a clear vision and expectations related to HIT for clinicians [39]. Users’ expectations are a considerable psychological factor that affects EHR adoption [40]. Leadership may need to develop expectations for staff accountabilities [39] to maintain HIT in good working order (eg, plug in workstations when not in use, report broken equipment).

### Timing of Adaptation to HIT

Adaptation to HIT over time may be explained in part by the Gartner Hype Cycle [41], which describes maturity and adoption of new technologies and applications. The Hype Cycle includes five phases: (1) innovation trigger, (2) peak of inflated expectations, (3) trough of disillusionment, (4) slope of enlightenment, and (5) plateau of productivity. The peak of nurses’ dissatisfaction occurred in R2, approximately nine months after HIT implementation. This finding may be due to nurses’ high expectations not being met at that time, corresponding with the Hype Cycle “trough of disillusionment.” Nurses began to grow impatient regarding supposed EHR benefits. Previous research indicates that nurses’ expectations for HIT include availability, speed, decreased work load, and ease of use [42]. In this study, nurses were able to provide suggestions and feedback to align HIT with their expectations regarding nursing workflow, training, and technology. However, the effectiveness of such communication is unclear. Research also indicates that nurses want to improve HIT through suggestions, but low communication levels and lack of feedback are barriers to enhancing system performance [43]. Variables related to nurses’ acceptance of EHRs include training/education, facilitating conditions, social influences, observability, and job relevance [44].

### Comparison of Nurses’ Experience With HIT With Other Clinicians

Previous research indicates that clinician satisfaction with HIT is mixed, which may be related to clinical documentation practice, workload, and productivity [45]. Physicians have reported dissatisfaction with template-based HIT documentation [45], possibly due to their high autonomy needs to prioritize work tasks [46]. In the current study, nurses also expressed dissatisfaction with template-based physical assessment because documentation was not streamlined. Nurses may be more accustomed to template-based documentation than physicians due to the use of standardized nursing care plans and clinical care pathways in the profession. However, if the HIT template does not match nurses’ mental model for accomplishing tasks, they will be dissatisfied.

Physicians and nurses may differ in perceptions of productivity after HIT implementation. While physicians may experience better productivity due to increased charges, improved work relative value units, and less time writing orders [45], nurses may not experience HIT value because there is no direct link in billing systems between individual nurses and patients [47]. Nurses’ productivity (hours of nursing care per patient day) is built into hospital room and board charges, so HIT may not be able to capture the work value of individual users like nurses [47]. Conversely, nurses’ experience with HIT may be similar to other clinicians’ experience. A qualitative study that aimed to understand physicians’ and nurses’ experience with EHRs found no major perceived differences based on profession [48]. A commonality among all professions appears to be the need for communication when implementing new HIT [46].

### Study Limitations

Generalizability of these findings is limited due to sampling nurses from one unit within an academic medical center. Self-selection bias may have occurred from voluntary participation. The timing of interview rounds was based upon interviewer availability rather than change theory. Although we conducted 30 interviews, some nurses participated over multiple time periods and the group comprised a small sample size (n=19). Despite the small sample size, recurrence of similar themes across multiple individuals established information saturation and data quality.

### Conclusion

A longitudinal qualitative approach for studying HIT adaptation facilitated understanding of thematic relationships over time. Although thematic interconnectivity differed due to nurses’ concerns and satisfaction at different time points, some trends were noted. Nurses appeared the most dissatisfied in R2, but many sources of dissatisfaction may be rectified, such as new equipment, refined policies, and improved systems to focus on patient safety. Approximately 18 months after HIT implementation, nurses’ perceptions appeared more balanced, as indicated by more consistent thematic frequencies and weaker cooccurrences in the Gephi chart. Balanced thematic distribution and interconnectivity within Gephi charts may be a visual indicator of HIT adaptation progress. Future research is necessary to confirm if researchers can replicate these findings in other samples.

## Appendix

Multimedia Appendix 1 Semistructured interview guide.

Multimedia Appendix 2 Theme codebook and definitions.

Multimedia Appendix 3 Themes with quote examples.

## Abbreviations

BCMA: bar code medication administration
E: theme
EHR: electronic health record
HIT: health information technology
ICU: intensive care unit
IT: information technology
R: round (of data collection)
WOW: workstation on wheels
