# Supplementary material for: Nurses’ Experience With Health Information Technology: Longitudinal Qualitative Study
Source: JMIR Med Inform. 2018 Jun 26;6(2):e38. doi: 10.2196/medinform.8734 (PMC6043728; doi:10.2196/medinform.8734)
Supplement: Multimedia Appendix 1 [file medinform_v6i2e38_app1.pdf]

Appendix A. Semi-structured interview guide

1. What has your experience been with EHR/BCMA implementation?
2. What did you expect in the beginning? Where are your expectations from (peers, management, literature, etc.)?
3. How has EHR/BCMA met your expectations (or not)? Did your expectations influence adoption of EHR?
4. Discuss specific experience in:
   a. workflow
   b. medication administration process
   c. physical assessment
   d. nursing care
   e. documentation
   f. unit’s teamwork and teamwork within the hospital
   g. quality of care
   h. communication
   i. emergencies, codes, or response team calls
   j. patients and families
   k. errors, overrides
5. What is the worst part (or most frustrating aspect) of EHR? BCMA?
6. What is the best part of EHR? BCMA?
7. How long did it take you to get used to EHR?
8. Describe how you adapted to the system? How do people, time or personal motivation influence you?
9. What factors influenced your acceptance of EHR?
