# Supplementary material for: Nurses’ Experience With Health Information Technology: Longitudinal Qualitative Study
Source: JMIR Med Inform. 2018 Jun 26;6(2):e38. doi: 10.2196/medinform.8734 (PMC6043728; doi:10.2196/medinform.8734)
Supplement: Multimedia Appendix 2 [file medinform_v6i2e38_app2.pdf]

Multimedia Appendix 2 - Theme codebook and definitions.

**E1: Nurses' Interaction with HIT:** how easy the system was to use.
#1:Equipment: physical technology used with EHR including computers, scanners, and workstations on wheels (WOWs).
#2:System-Changing Layouts: IT professionals' modifications of the system interface.
#3:System-Errors due to Disorganized Layout: interface design contribution to documentation or navigational errors.
#4:System-Functionality: software capabilities and operational issues.
#5:System-Easy Navigation: ability to move through the system easily.
#6:System-Difficult Navigation: challenges regarding moving through the system.
**E2: Nursing Performance regarding Task Accomplishment:** nurses' workflow and effect on task performance.
#7:Documentation-Efficient: documentation tasks that were faster over time, or faster than paper charting.
#8:Documentation-General: nurses' notations within the patient health record.
#9:Documentation-Inefficient: documentation tasks that were slower over time, or slower than paper charting.
#10:Documentation-New Features: modifications of EHR functionalities over time.
#11:Documentation-Not Streamlined: records not organized according to nurses' mental model.
#12: Documentation-Streamlined: records organized within nurses' mental model.
#13:Documentation-Thoroughness: comprehensiveness of electronic health record (compared to paper charting).
#14:Environment: the physical space including rooms, hallways, or doorways.
#15:Feedback on Performance: how HIT influenced nurses' actions and tasks through auditory/visual cues, or peer advice.
#16:Impact on Workflow: the effect of HIT on the sequence of operations to complete nursing tasks.
#17:Med Admin-General: nurses dispensing drugs or medicines to patients.
#18:Med Admin-Timing: overdue or early medication administration and documentation.
#19:Med Admin-Overriding and Linking: administering a medication before pharmacist review, and the need to connect the administered dose to a medication order.
#20:Physical Assessment: examination of a patient's anatomic findings.
#21:Rare Events-Blood Admin: the task of blood administration.
#22:Rare Events-Emergency: tasks associated with emergency care, such as code blue documentation.
#23:Rare Events-General: infrequent nursing tasks, excluding blood admin and emergencies.
**E3: Unit-specific Teamwork**: communication and collaboration between nurses, patient care assistants, and nursing managers within the nursing unit/department.
#24:Nursing Unit Collaboration: nursing staff working together to operate HIT.
**E4: Interdisciplinary Teamwork:** collaboration across disciplines or departments.
#25:Communication across Disciplines: exchange of information between departments or units.
#26:Missed Communication: omitted, unclear, or inadequate patient information between departments or units.
#27:Patient Transfers: movement of the patient from place A to place B.
#28: Shifting Responsibility: when nurses completed tasks that fall under another person or department's duty.
#29:Unequal Standards: varying expectations regarding communication and documentation between departments or nursing units.
**E5: Quality of Care**: HIT effects on error reduction, patient/nurse satisfaction and better care.
#30:Better Care: how HIT improved health care services for the benefit of a patient.
#31:Error Reduction: perceived decrease of medication errors and/or risk of patient harm.
#32:Negative Impact on Care: ways that the system limited or hurt patient care.
#33:No Change in Care: neutral effects of HIT on patient care.
#34:Nurses' Dissatisfaction: how HIT did not meet nurses' expectations and their disappointment with the system, workflow, and outcomes.
#35:Nurses' Expectations: anticipated benefits of HIT and how nurses imagined it would influence care or patient outcomes.
#36:Nurses' Satisfaction: nurses' approval of the system, workflow, and outcomes.
#37:Patient Experience and Satisfaction: patient interaction or perception of the EHR or BCMA.
#38:Patient Experience-Modern Healthcare: patients' perceptions regarding the innovation of HIT.
#39:Patient Experience-Patients Adjust: how patients adapted to the HIT environment.
#40:Patient Safety: how EHR or BCMA prevented errors, injuries, or accidents from reaching patients.
#41:Patient Safety-Catching Deterioration: trending data in EHR to allow nurses to detect abnormalities that could lead to

patient decline.
#42:Potential for Error: mistakes that could still occur with HIT.
**Adaptation Factors**: elements that affected HIT acclimatization including internal resources (individual technology proficiency) and external resources (organizational support).
#43:Clinician Involvement with Design: the desire for bedside clinicians to plan HIT for real-world use.
#44:EHR Playground: a non-active version of the EHR where nurses practiced using the system before implementation.
#45:IT Support Team: IT professionals that addressed and solved equipment and system issues in the clinical environment.
#46:Self-learning: acquiring HIT knowledge and skills on their own time or with real-world application.
#47:Staffing: number of patients assigned to nurses.
#48:Super Users: nursing peers on each unit that were trained to calm anxiety and share hints, tips, and techniques on HIT.
#49:Technology Proficiency: individual nurses' comfort and skill with computers that affected adaptation to the system (typing, etc.).
#50:Training: employer-provided education, classes, or materials to promote adaptation to HIT.
#51:Users Adapt: nurses' adjustments to learning tasks, navigation, and the HIT system.
**Organizational Factors**: communication or HIT decisions made by leaders that influenced nursing work.
#52:Leadership: how management or hospital administration influenced users' adaptation to HIT.
#53: Policies: principles, rules and guidelines developed for decision making and actions related to HIT.
#54:Suggestions for Improvement: ideas and proposed changes to enhance the EHR, hospital policies, or workflow.
