# Supplementary material for: Nurses’ Experience With Health Information Technology: Longitudinal Qualitative Study
Source: JMIR Med Inform. 2018 Jun 26;6(2):e38. doi: 10.2196/medinform.8734 (PMC6043728; doi:10.2196/medinform.8734)
Supplement: Multimedia Appendix 3 [file medinform_v6i2e38_app3.pdf]

Appendix B – Themes with quote examples

*Appendix B details nurses' quotes separated by theme from all time points. The #number corresponds to Table 1 and the Gephi figure labels. The parentheses after themes indicate the frequency of themes in all rounds combined. The parentheses after each quote contain when (Round number, i.e. R) it was said and who (Participant number, i.e. P) said it.

**E1: Nurses' Interaction with HIT**
#1:Equipment (105)
"What I don't like is how loud they [the workstations on wheels] are and how big and bulky, the drawbacks. I mean the only good thing about that is I don't have to knock as much because they can hear me like rumbling through." (R1 P3)<br>*"I do like that we are getting wireless [scanners], sometimes it can be frustrating when it's connected to the WOW and the patient's across the room and there's like obstacles and I'm like, I can't reach you. So the wireless [scanners] are nice." (R3 P1)*
#2:System Changing Layouts (5)
"There are a lot of changes, it always is constantly changing. And I know that the idea of it is to make it a better system. But the changes always come with a price, as far as not knowing if you're doing things right or not for a while." (R3 P8)
#3:System Errors due to Disorganized Layouts (5)
"When you have to click on like the dropdown boxes when you click on stuff. Some of the choices, they'll have opposites like right next to each other. Or I know they had cooperative and combative like right next to each other... And it's easy to just click on the wrong one." (R3 P4)
#4:System Functionality (45)
"They [the workstations on wheels] like log you out, and then you have to reconnect to EHR somehow, and that takes forever. And you're sitting there waiting like, come on, pop up screen so I can log in to give medicine. Because the person is like screaming, and you're yelling come on. And it takes like a couple of minutes to really get it open and logged back in." (R2 P7)<br>*"There are [medication] barcodes that don't scan. And we always get told in the staff meetings, 'This person had 100% scanning,' and I gave like 400 more meds than they did. You just get irritated. You're like, I swear I scanned it, and it never showed up." (R3 P9)*
#5:System Easy Navigation (20)
"It's nice how it's organized and that you can go through each tab and find it quickly, ask the questions and be done with it. And it's right there, versus the old way…you kind of had to remember what you were asking the patients and then remember what they said and then go and chart everything at once." (R1 P10)<br>*"You don't have to flip back and forth from your documentation to the order set, so that's a little bit easier in that aspect." (R2 P11)*<br>*"It's [EHR] much easier, more user friendly now. It's even easier to navigate…They've changed a lot of things. More of the nursing flow sheets that have made it easier for nurses. So it's much better than a year ago." (R3 P1)*
#6:System Difficult Navigation (40)
"It's just hard to go back in and find something, find out where something is... Like a specialty bed, it's hard to go back in and try to figure out. It's like specialty beds are like under skincare. So, we're just trying to get used to it." (R1 P8)<br>*"It's frustrating that you can't find the rows you want to and that you have to flip back and forth through your tabs and everything can't be in a clean concise manner." (R2 P3)*

**E2: Nursing Performance regarding Task Accomplishment**
#7:Documentation Efficient (25)
“I think it saves time because you’re verifying the med at the bedside as well as charting that the med was given. So, it saves that extra step that we had to do when we did paper charting. So, for the most part I think it’s a good tool.” (R2 P8)<br>*“Charting, obviously, I think has gotten a little more proficient. It takes me a little less time.” (R3 P9)*
#8:Documentation General (25)
“People are just, like I said, I think frustrated because they have more charting to do and in less time to do it.” (R2 P2)<br>*“Until you get that [charting] done, it's lingering over you the whole shift, people are asking about different things, and it's not in the computer. Or you feel like you've done all this work and there is no proof of it yet.” (R3 P9)*
#9:Documentation Inefficient (56)
“Med pass takes a little bit longer. I've noticed that when people actually do have a chance to chart they are rushing through it.” (R1 P5)<br>*“And EHR, as far as documentation goes, is horrible. Everybody complains about it. It adds at least an hour to two of extra charting.” (R2 P2)*
#10:Documentation New Features (8)
“They now have like a book mark so if you have to leave the computer or have to log out, you can put book marks where you last were at, so you can straight to it, so you don't have to [search]…Where was I, I don't remember where I was at in my charting and have to search through.” (R3 P1)
#11:Documentation Not Streamlined (49)
“I’m not a huge fan. I think it’s [EHR] really, really cumbersome and complicated. And I think it could’ve been much more simplified and easier on the eyes. It seems like it just makes your eyes want to bleed at the end of the day, after looking at it all day. It’s just a lot going on.” (R1 P6)<br>*“I think a lot of things don’t get charted in because it does take a lot of time still to find things…I think too that you might miss charting things because you're doing the assessment, and then you go to the computer. Where before when we would write things down, we actually did write from head to toe.” (R2 P10)*
#12:Documentation Streamlined (22)
“Once you scan the patients and scan the drug…it’s taking care of your five checks and then it’s done. You…don’t have to go back to the old system at the nurses’ station and remember the time that you gave it…I like that.” (R1 P3)<br>*“It is easier to chart, to go through it this time than before. Because I think they kind of edited it [EHR] and it's better now, it's more compact, more precise.” (R3 P10)*
#13:Documentation Thoroughness (29)
“I feel like I’ve done a more thorough job in my day and knowing that…my hand is being held on what to do… which makes it nice…you’ll forget to do a little assessment. But you’re prompted on the charting through the EHR to do it; whereas with the old system, it seemed really generic or like more on actual documentation writing. You know documentation to catch all the points that are important.” (R1 P3)<br>*“With the pen and paper, like you just wrote it down real quick and then you were done. But, I feel that I probably missed more with the paper charting, where now it [EHR] holds me responsible for actually taking credit for like the education pieces.” (R2 P11)*

#14:Environment (15)
"Your back is to the patient 90% of the time during your med-pass or when you're looking up your meds because you can't, the way the rooms are situated, you can't put the computer like up towards the head of the bed because there's usually a chair there or their bedside table. So you kind of back in and then you have your computer there. And they're like laying in a bed behind you." (R1 P6)<br>*"If I were a patient, I would think our hallways look atrocious. They look like a war zone. Not only do you have your original isolation carts, everything out in the hallway, but now you have like 700 computers trying to be plugged in in different ways. A patient is trying to come down the hall, the family member is coming, I'm trying to drive my WOW in between you guys because this patient has been asking for pain meds for 10 minutes. I think it just looks messy. And I don't really know a way to eliminate that, because it's not really that it is messy, it's just cluttered." (R3 P9)*
#15:Feedback on Performance (50)
"Right now EHR will tell you that you [missed something]…because there's a list of tasks that are due. And they go from white to yellow, to red when they get overdue. If it's a little bit overdue it's white. If it's very overdue, it's yellow. And if it's way overdue, it's red…It's going to ding you and tell you that something's missing…some documentation is missing." (R1 P4)<br>*"Yeah, we're doing peer audits where they're looking through our charting. And I got good feedback. The only thing I was told was that I was documenting too much." (R3 P8)*
#16:Impact on Workflow (104)
"Right now I don't see any improvement in the workflow. In fact, EHR is taking too much time and taking away from patient care." (R1 P5)<br>*"We're having to spend all this more time literally charting the same exact thing we just charted. It's silly. So, that effects work flow a lot. And, it affects morale and it affects nurse's stress levels. It's frustrating when you're re-copying things just because somebody in management says that's what we should do." (R2 P2)*<br>*"I come out of the patient's room and do it [physical assessment]. My admission things where you have to ask them all those questions, now that is in there and do, because I can't, I wouldn't remember all of that. So that I sit in there and go down the list for list and do that. But the assessment, I don't do that. I tend to go to nurses' station or pull my WOW out of the room. And you can sit in the hallway at night and do it." (R3 P6)*
#17:Med Admin General (43)
"For me the best part is the Medication Administration. That, to me, is like the gold standard of EHR." (R2 P8)<br>*"Med passes are a lot safer now. And I notice that because in my other job where we don't have it, there's that comfort of knowing that EHR is looking over your shoulder as you're giving the meds by scanning. It gives you peace of mind. You feel that you're really being safe." (R3 P8)*
#18:Med Admin Timing (15)
"I just chart them [medications] if they're late. I don't know if can do anything else. I just do it when I have to. I mean there is not much you can do, otherwise." (R2 P10)
#19:Med Admin Overriding and Linking (26)
"The override pull things are very annoying and very difficult to understand. I think half the time I just click until it finally accepts what I want it to do." (R2 P5)<br>*"Overrides are a pain in the butt, linking them, making sure they're linked right. The problem is if somebody before you overrode for it and then doesn't link for it correctly, then you almost have to link theirs before you can link your own." (R3 P9)*

#20:Physical Assessment (38)
"It's just the assessments are so horrid to chart." (R1 P6)<br>*"In a way it's [EHR] actually made my assessments more thorough. Like there were points in a GU assessment that without having to read it every day I might not have paid as much attention to it. So I feel like it's actually benefited me in my assessment skills." (R3 P8)*
#21:Rare Events Blood Admin (16)
"There was a day, Sunday, that I worked and I had to hang blood and they had just changed the blood administration flowsheet and I had not transfused blood since they made the change. And, nobody in my unit knew what to do so my charge nurse had to call another charge nurse from another unit to come and help us." (R2 P8)<br>*"It's very confusing. And in the beginning, they put the cheat-sheet on the blood bag, which was easy. We're like oh, here it is, here it is. Well then they stopped doing that, because you know, everyone should know how to do it by now. But if you don't get blood for a month or something, then you don't know how to do it." (R3 P5)*
#22:Rare Events Emergency (45)
"For a lot of people it's because we don't use it all the time, it's really difficult. I mean even as a super user it's taken me like three or four times doing it [emergency documentation] just to try to get comfortable." (R1 P10)<br>*"It's [emergency documentation] very complicated and it's very busy on the screen and if you don't do it every day it's really hard to remember. You have to ask every doctor what their name is and type it in and anybody in the room. And then, click that they're in the room and if they leave, click that they're out. Plus, we're supposed to document all the things going on and it's pretty insane. The STAT nurses help a little bit but they even aren't real comfortable and they're the ones that are in codes all day every day." (R2 P2)*
#23:Rare Events General (4)
"If we get an off-service patient, finding where you chart certain things that you know you're supposed to chart that we don't do on a regular basis is hard." (R2 P5)
**E3: Unit-specific Teamwork (68)**
#24:Nursing Unit Collaboration (24)
"At the point that you have to do blood, by the time, you forget what the whole process was. So, you go back and say, "Hey, you gave blood yesterday. How did you do this?" So, I think it [EHR] has brought us closer together. It has made us work closer together." (R2 P8)<br>*"I personally think that we have a great team. So we've always helped, worked together and helped each other. So we do help each other a lot with the charting. If we can't find something, we'll ask somebody. So we work great together." (R3 P2)*
**E4: Interdisciplinary Teamwork**
#25:Communication across Disciplines (54)
"The best part I think is that it's all legible and it's timed and signed by someone who has a name and you know who it was and when they did it. Whether that's nurses or pharmacy or lab or doctors." (R2 P1)<br>*"I mean it's [EHR] great. Compared to paper charting and reading handwriting, oh my gosh; like the notes from the doctors, the H & Ps, it's great. And then their scanning their consents in there so you can see before surgery and all that. So I think it works well. I don't feel like we're passing papers around or anything anymore." (R3 P5)*

#26:Missed Communication (5)
"If I have communication or progress notes, I'll write something, and I don't know who's reading it. I always feel if I document it that I can't guarantee … I still would have to call the physician about a situation and not rely on my progress note that I put in to relay information." (R3 P8)
#27:Patient Transfers (34)
"When we get ED…we can go and look at, before they come up, we can access their records, you know. So that makes it nice. You can have a general idea on what's coming before you even talk to the nurse and get report." (R2 P4)<br>*"We try when we're getting a patient, the best thing to do is look at the patient's information. So when they're giving you report, you can ask questions on things. Is that blood pressure corrected? Did they get that IV antibiotic, you know? And you can have a better report." (R3 P6)*
#28:Shifting Responsibility (16)
"That's what physicians alone, like putting orders in, they're like well, you're right by the computer, go ahead and change it for me. And it's like I can do it, but I don't want to do it, because it's not my job." (R2 P11)<br>*"Not everyone follows-through with that and document everything they need to document on there. So if the patient is getting two units of blood and first shift didn't do everything, in order to make it work for the next bag, you need to go through and correct a lot of things that they did." (R3 P6)*
#29:Unequal Standards (41)
"I know like with our docs that we have up on our floor a lot. The primary service… they are so resistant to pushing a computer around and instantly putting orders in as they go around patient to patient. So then it's like they round on all their patients, then they go down to the ICU and then they come back up. And then they'll put their orders in." (R2 P11)<br>*"It's a lot to do when you have four or five patients on a busy med-surg floor. I know in like ICU's and stuff they have a lot more to chart sometimes. But it's very patient focused, it's very centered. You go do your thing, you can sit down and watch your patient, and chart. I have five patients; I cannot sit down until 2:00 o'clock and chart my 9:00 am assessments, because I haven't gotten the time. I've got to travel." (R3 P9)*
**E5: Quality of Care**
#30:Better Care (37)
"I'd say that makes you focus more on patient care. You know silly little things like, when was your last bowel movement. You need to fill that in. And, sometimes when you look you see, OK, today is the 20th. Wow, last time he had a bowel movement was the 10th. OK. So, yes it [EHR] makes you focus in more." (R2 P6)<br>*"You have everything, all your answers that, well pretty much all the answers that you need right in front of you. There's notifications for stat orders, new orders, out of range values, so I would say that the quality of care for patients is much better." (R3 P1)*
#31:Error Reduction (57)
"There have been several times when I was scanning when it [EHR] clued me in to, oh, that's a half a pill or something like that, that I hopefully would have caught beforehand, but it was an extra reminder." (R1 P1)<br>*"The triple-check… Because…you check your medications, you check the right patient, but then having that extra backup of scanning everything and being like it's not the right time or oh, that's the wrong dose." (R3 P7)*

#32:Negative Impact on Care (34)
"I think that we don't have that like patient interaction as much, because we're like staring at the computer screen when we're in their room. Whether it's looking at their vitals…reading the MAR [medication administration record], clicking it, putting in all our million different passcodes we have to put in...You scan the patients and then immediately you go back to the computer to see if it scanned right, if you got the checkmark. So your focus is the computer, when your focus should be on your patient." (R1 P6)<br>*"I think it [EHR] has affected quality of care negatively because EHR is so labor intensive that you, well, me, I try to rush my assessment and consolidate my patient care so I have time to sit and chart. Because, charting is now the biggest part of your day and that, I think, it should be the other way around. Patient care and then your charting. But, we spend more time charting than we spend doing actual patient care." (R2 P8)*
#33:No Change in Care (2)
"It's a give and take. I think it's [patient care] probably improved in certain areas and down in other areas." (R2 P5)
#34:Nurses' Dissatisfaction (120)
"I feel that it's [EHR] more a hindrance, because in the system we can't chart and get it across what is happening with our patients…right now I think we're at a disadvantage with EHR than we are anything else." (R1 P5)<br>*"Constantly having to have a computer with you. You feel like you're attached to it. And sometimes, especially when a patient first gets there, I feel like I'm typing… I'm trying to get questions answered and talk to them…you're staring at this computer screen rather than like talking and having a conversation with them, because you're trying to answer the questions while they're talking to you…I'm really not paying much attention to them because I'm staring at this computer back and forth." (R2 P7)*<br>*"I hated EHR. [Laughter.] I don't know. A good three months. I struggled. I struggled. When we first started we had 3 patients and I never left work until about 8:00 every night…I thought, as a charting tool it left a lot to be desired, so I didn't really like it. But, they've made improvements, they've tried to make the interface a little more nurse friendly. I appreciate that but I still think it's got a long way to go." (R2 P8)*<br>*"The computers never work. It's like you can't load it up, your scanner doesn't reach, it's not scanning. I think people most get frustrated with dealing with the actual equipment itself than the actual system." (R3 P9)*
#35:Nurses' Expectations (28)
"I thought it [EHR] was supposed to be quicker for pharmacy to get our meds and get those orders in, but I haven't really noticed any speed there as far as from before." (R1 P1)<br>*"I just expected this super-program that right off the bat was going to be excellent. And for a long time, I felt like we were the beta testers. It was like, this isn't working now. Or, maybe in six months, this will work." (R2 P3)*<br>*"I expected EHR would simplify my work and allow me more down time, well, not really down time, but more time for patient care. But, it has proven to be the opposite. I spend more time trying to figure out EHR and my documentation and I cut my patient care short. And, I don't like that because I want to focus on patient care." (R2 P8)*

#36:Nurses' Satisfaction (85)
"I think generally speaking, I think the nurses are pretty happy with it [EHR]…having that change, I think there's that resistance to it but I think people see the benefit of it [EHR]." (R1 P10)<br>*"It does get better and that's what our nurse manager had told us. You know, at first it's [EHR] intimidating, but you're actually going to like it [EHR]. They swore to us that we were going to like it. [laughter] And I do. Like I said, I really do, it just takes time." (R2 P10)*<br>*"I've gotten to like it [EHR]. It's become a little more user-friendly and some substantial changes have been made that kind of facilitate your nursing workflow. So my experience now is a lot better than when it first got rolled out." (R3 P3)*
#37:Patient Experience and Satisfaction (82)
"It may give them a little more confidence in knowing that there are checks, you know, safety checks for them. You know, I get a lot of jokes all the time about them feeling like groceries and stuff like that." (R2 P4)<br>*"You have information right at your fingertips. And I think patients; patients get empowered when they have information. So to me, it's [EHR] been a good experience for the patients." (R3 P3)*
#38:Patient Experience Modern Healthcare (4)
"I think people when they see like technology, they think, "Oh, it's like a fancy hospital." They do all computers. Exactly, so yes, that's fine." (R1 P6)
#39:Patient Experience Patients Adjust (4)
"At first they were a little…not very friendly about it, because they were being put off. Because we were so busy, so busy with the computer, but now, I mean pretty much everybody knows you know, give me your arm, do this, you know." (R1 P8)<br>*"It's just a matter of telling the patient way ahead of time what they're going to be expecting in the middle of the night. So if you kind of tell them about it, then they have that in mind, oh, she's going to wake me up [to scan]. And then when you wake them up at that time they're not going to be that grumpy or stuff like that." (R3 P10)*
#40:Patient Safety (40)
"Even if we have two Mr. Smiths because of the whole scanning it's almost, I shouldn't say foolproof, but you've got to try really hard to mess up your meds." (R2 P6)<br>*"The safety net. That absolutely is the best. I feel like I'm a safer nurse by knowing that I'm giving the right med at the right time." (R3 P8)*
#41:Patient Safety Catching Deterioration (5)
"I added a column so I can see what patient's MEWS [modified early warning score] scores are because if they're over a certain number, we need to be looking into why it's high and see if we need the ERT [emergency response team] or get the patient off unit." (R3 P1)
#42:Potential for Error (52)
"You could get in a habit of only relying on the computer, like not even looking at your medications…. But if the order is put in wrong and the medication you get out matches that wrong order, then you don't even know you made a med error." (R1 P6)<br>*"I know this sounds bad, but you'll hear nurses being like clicking, "Oh, my gosh, I forgot what patient I was even charting on." Because you're just so used to saying the same things. And unless you have a really intense patient, a lot of people are breathing just fine, a lot of people are urinating just fine. And you're just sort of clicking, and clicking, before you even realize, oh, wait; I'm charting on him not her. Or, I don't even know what I just charted." (R3 P9)*

**Adaptation Factors**
#43:Clinician Involvement with Design (14)
"I just think the different floors need to get together, now that they've seen EHR, they just need to get together and see what's the most common on their floor and be able to structure it so it fits their floor." (R1 P5)<br>*"Just having like the actual staff nurses that work in that facility help to make the charting or develop it [EHR], would be useful, because again, people who are computer programmers and people who were nurses and are just hired by a tech company, don't really know the real workflow of what's going on." (R3 P7)*
#44:EHR Playground (12)
"[The playground] was somewhat helpful for me to get an overview of EHR. But…having not used it and really not knowing how it's going to function, there was a lot I just didn't know. I just couldn't experience until we actually went live with the system." (R1 P10)<br>*"Give more opportunities actually with an EHR playground, I think that would have been very helpful. If you have to go spend some time on the EHR playground, it may have helped people prepare for it. Doing more like demos and having someone say, "Hey, we're going to get this admission, and we're going to show you how to release orders," and then have people do that. Like real applications, things that we're going to see on our floor." (R3 P8)*
#45:IT Support Team (33)
"It was actually really, really helpful. The lady [from IT] actually did a really good job of helping me out, figuring out the solution to my problem. But really even the helpdesk, it can be kind of hard and time-consuming…I don't know what their use of EHR is, other than trying to help us out. But, you know you feel like it can be hard to communicate to them from a nursing perspective, our needs." (R1 P10)<br>*"We're supposed to call the helpdesk. But, I just feel like I don't have time to call the helpdesk. If I have a computer that's not working, I just go find another computer." (R1 P6)*<br>*"Only if I get locked out and things like that. I don't call them [IT] for simple questions anymore. We ask each other." (R2 P10)*
#46:Self-learning (19)
"Just a lot of, you know, applying the program and digging into it and looking at all of the different tools you can use. I mean that's how I, I mean I found that to be interesting. I've learned a lot just by doing that." (R2 P4)<br>*"They wanted us to like come in on our off time and practice and things. Like no one wants to do that. You're already there. You're already stressed about it. The last thing you want to do is stay late after work and practice more of it." (R3 P5)*
#47:Staffing (30)
"I think they went back to regular staffing grids too soon. Because, for example, last night I had three high acuity patients, and I can tell you with the system how it is, there was charting missed on them. Like the early rounding, and how they want the different hours, how many hours for IV assessments and all that." (R1 P5)<br>*"We still at this point have like one patient less than we did before. So we're starting to get to the point where it's possible to go up and have another patient, if that's what the ratio calls for at the time. But yeah, I loved the fact that we started very slow and then started adding patients. That was very helpful." (R3 P2)*

#48:Super Users (30)
"Having the super users was the best idea I think they could have had." (R2 P1)<br>*"It wasn't just some strangers. They [Super Users] were our coworkers, which made us more, it seemed more approachable, you know, not feeling like you're dumb or having to worry about… So that was nice, knowing that your coworkers are going to be the ones helping you." (R2 P4)*<br>*"The super-users, that's a must have, because you don't have time to sit down and look through a handout or a booklet to see how to do something. You need it now. And so to have someone right there was huge." (R3 P5)*
#49:Technology Proficiency (25)
"See I'm a dinosaur to this. With computers, I'm not as computer literate like a lot of the younger ones are. So, it takes me a little longer to find things." (R1 P8)<br>*"The classes were real intimidating, when we went to the classes. I mean we had people going out crying because it was so…if you didn't catch on, because they were moving so quickly, and we were a little bit on the slower side, that it was harder." (R2 P10)*
#50:Training (44)
"They made us all nervous and we had to go through so much training and it was so far away, like it was months before we actually went live…I felt like we forgot some of that stuff in that timeframe. So, it was a lot to do, you know all the training and it was like four-hour sessions. So it was like crammed so much stuff into those short little timeframes. And then it didn't correlate to real life. Like they taught us stuff and they taught us how to do assessments and stuff. But, the stuff that we really do, they didn't spend a whole lot of time on." (R2 P11)<br>*"We had four sessions of I think four one-hour training sessions. I just think it was rushed. I understand that it's a huge enterprise to roll this out. And so you've got to what's best for the company, but I think some people got left behind." (R3 P3)*
#51:Users Adapt (61)
"We're getting there. I mean, it's a start. EHR is a start. You have to crawl before you can walk and we're kind of crawling with our technology slowly, but we're getting there. I think we're going to get there." (R2 P6)<br>*"I think initially EHR; we were more charters than nurses, when we first rolled out EHR, because the charting was so time-consuming, so labor-intensive. But I think the novelty has worn off and we're becoming proficient. And we can really spend more time with the patient. And then once you become proficient, it just, that's when you see the improvements." (R3 P3)*<br>*"Don't expect to get it all in a month. Give it a few months. At first it [EHR] will seem like your enemy and a burden or hindrance. But as time goes by you're going to find it to be a good thing for your career, for your job." (R3 P8)*
**Organizational Factors**
#52: Leadership (11)
"They [IT] send emails and we have that one source. But eventually, after so many emails, you just go, oh, my God. So really if there is a major change affecting us, our managers let us know, like you need to chart this differently or do this differently." (R2 P5)<br>*"I mean they [leadership] were very supportive of us. I mean providing answers to questions and resources. Just be patient with your nurses. And always use super-users. Just have the confidence that even if it's going to be rough at first, that the staff is going to, they're going to be able to do it. Everything is going to get smooth eventually." (R3 P2)*

#53:Policies (61)
"We really wanted to get the copy [and paste] over for the assessment that they were going to let us do it and right they even gave them, they gave in-service and everything to the managers and stuff and said it was going to happen and at the last minute, they said no." (R2 P1)<br>*"We're supposed to chart in real time after it happens. I couldn't tell you one person that does." (R3 P9)*<br>*"Them adding every other day something new. If you add something, take something away, but they're not. So that part is annoying… They keep adding and take nothing away." (R3 P9)*<br>*"But, I feel like we don't see results that we think are great ideas fast enough, if ever. We have been saying forever, where's a copy button if we're going to do these two assessments? Where is this? And they wouldn't give it to us, they wouldn't do these things. If you can cater it so easily to us, as it was advertised, why is it so difficult to make these changes that I really think would help my workflow as a nurse? Or something of that nature, I feel like it's not there. Or they think they're helping us and I'm like, really? That's probably not the best way you could have done it." (R3 P9)*
#54:Suggestions for Improvement (56)
"For example, maybe a patient has an A-line [arterial] in PACU [post-anesthesia care unit] that they remove. It would be nice just not to see it there after it's removed. Or, if I want to look it up, I could look it up." (R1 P10)<br>*"I would like any time you open up a patient's chart, it automatically defaults to that time." (R1 P10)*<br>*"It [EHR] needs like a glossary or something where you can…type in this[word], and then it should be able to tell you where to find it." (R1 P8)*<br>*"It would be probably a little easier to have a workstation in every room. You don't have to worry about dropping stuff in there or the mouse coming unplugged, and then you can't plug it back in, but…the other downside to having a computer in every room is logging in, four to five different times just to give medications, where when you have your workstation on wheels, you're logged in; you just go." (R1 P11)*<br>*"Wireless everything, so we don't have more wires to drag around would be wonderful. And not have to click to so many tabs to chart everything." (R2 P7)*<br>*"If I wanted to communicate to a physician or a doctor that way, knowing I would see "Read by Dr. So-and-So at this time," that would be great." (R3 P8)*
